# 自噬在EGFR-TKI类肿瘤靶向药物对肺癌的治疗和耐药中作用的研究进展

**DOI:** 10.3779/j.issn.1009-3419.2016.09.09

**Published:** 2016-09-20

**Authors:** 其程 张, 克 徐

**Affiliations:** 300052 天津，天津医科大学总医院，天津市肺癌研究所，天津市肺癌转移与肿瘤微环境重点实验室 Tianjin Key Laboratory of Lung Cancer Metastasis and Tumor Microenvironment, Tianjin Lung Cancer Institute, Tianjin Medical University General Hospital, Tianjin 300052, China

**Keywords:** 表皮生长因子受体激酶抑制剂, 自噬, 耐药性, 肺肿瘤, Epidermal growth factor receptor-tyrosine kinase inhibitor (EGFR-TKI), Autophagy, Drug resistance, Lung neoplasms

## Abstract

表皮生长因子受体激酶抑制剂（epidermal growth factor receptor-tyrosine kinase inhibitor, EGFR-TKI）是一类针对肿瘤细胞中EGFR的异常活化而开发的肿瘤靶向药物，可以有效抑制带有*EGFR*敏感突变的肿瘤细胞的生长。然而先天性以及获得性耐药严重制约了该类药物的使用。近些年的研究发现自噬（autophagy），作为一个细胞编码的高度保守的应对压力的存活机制，其与肿瘤的发生发展及抗肿瘤药物的耐药密切相关。EGFR的激活可以通过多条通路调控自噬。EGFR-TKI也可以诱导自噬，且自噬在EGFR-TKI的治疗和产生耐药性的过程中发挥着双刃剑的作用：一方面EGFR-TKI诱导的自噬是肿瘤细胞的一个保护机制，联合使用自噬抑制剂可以增强药物的细胞毒性效果；同时还有研究证明EGFR-TKI诱导的高水平自噬可以在凋亡缺陷的细胞中造成自噬性死亡，这种情况下联合使用自噬诱导剂则可能产生更好的效果。因此，针对不同的情况通过调控自噬以提高EGFR-TKI的治疗效果是一个颇具前景的治疗方案。本文对EGFR-TKI和自噬相关的信号通路进行了阐述，并对自噬在EGFR-TKI类药物对肺癌的治疗和耐药中作用的最新研究进展进行了总结，为设计联合方案提高EGFR-TKI的抑制效果，降低耐药性提供线索。

## Abstract (en)

Epidermal growth factor receptor tyrosine kinase inhibitor (EGFR-TKI) is a group of targeted-drugs which effectively inhibits the growth of tumor cells with sensitive mutations in *EGFR*. However, the innate and acquired resistance are major obstacles of the efficiency. Autophagy is a highly conserved self-digesting process in cells, which is considered to be associated with cancer development andchemoresistance. The activation of EGFR may regulate autophagy through multiple signal pathways. EGFR-TKIs can induce autophagy, however, the function of the inducted autophagy remains biphasic. On one hand, autophagy induced by EGFR-TKI acts as a cytoprotective response in cancer cells, and autophagy inhibitors can enhance the cytotoxic effects of EGFR-TKI. On the other hand, a high level of autophagy after treatment of EGFR-TKI can also result in autophagic cell death lacking features of apoptosis, and the combination of EGFR-TKI with autophagy inducer might be beneficial. Thus, autophagy regulation represents a promising approach for improving the efficiency of EGFR-TKI in the treatment of cancer patients. Here we summarized the signaling pathways involved in EGFR-TKI induced autophagy, and reviewed the roles of autophagy in the treatment and chemoresistance of EGFR-TKI treatment in lung cancer.

肺癌是全世界最常见的恶性肿瘤之一，也是发病率和死亡率增长最快、对人类健康和生命安全威胁最大的恶性肿瘤。目前，男性中肺癌的发病率和死亡率在各类癌症中均位列首位，女性中其发病率列第二位，死亡率居第一位。肺癌已经成为人类的第一杀手^[1]^。根据组织学特征，肺癌可以分为两个亚型：小细胞肺癌（small cell lung cancer, SCLC）和非小细胞肺癌（non-small cell lung cancer, NSCLC）。NSCLC约占总数的80%-85%，可分为3个类型：腺癌（adenocarcinoma）、鳞癌（squamous cell carcinoma）和大细胞癌（large cell carcinoma），其患者的5年存活率只有约15%^[2]^。随着对肿瘤发生机制研究的不断深入，根据不同的“癌基因依赖”（oncogene addiction）现象^[3]^，现在可以对不同组织类型的肺癌进行进一步的分子分型，并实现靶向治疗。对带有表皮生长因子受体（epidermal growth factor receptor, *EGFR*）敏感突变的NSCLC患者，可以使用EGFR酪氨酸激酶抑制剂（EGFR-tyrosine kinase inhibitor, EGFR-TKI）单药实现有效的治疗。其中厄洛替尼（erlotinib）已经成为带有*EGFR*敏感突变的NSCLC治疗的一线方案。然而在临床中发现，随着治疗进程的进行，绝大多数患者都会出现明显的耐药现象，这也是EGFR-TKI等靶向药物治疗的最大问题。自噬（autophagy）是细胞自身编码的应对营养缺乏等环境压力的过程，其由一系列蛋白参与和调控，并与多种细胞过程和反应有着密切的联系。近些年大量的研究发现自噬在肿瘤的发生和治疗过程中发挥着“双刃剑”的作用：一方面作为一种肿瘤细胞面对压力时的存活机制，起到保护细胞的作用；另一方面，自噬性死亡在某些情况下对杀伤肿瘤细胞起着关键作用。因此，针对不同情况联合使用自噬调控药物，被认为是提高肿瘤靶向药物治疗效果、降低耐药性的关键调控点。

## 1 肺癌EGFR-TKI类靶向药物及耐药机制

### 1.1 EGFR-TKI类靶向药物

EGFR是表皮生长因子（epidermal growth factor, EGF）引起细胞增殖和信号传导的受体，属于ErbB受体家族，该家族包括EGFR（HER1或ErbB-1）、HER2（ErbB-2）、HER3（ErbB-3）和HER4（ErbB-4）。EGFR是细胞表面的一类受体酪氨酸激酶受体，可被胞外生长因子如EGF或TGF-α（transforming growth factor α）等激活^[4]^。与配体结合后，EGFR由单体转化为二聚体或与ErbB受体家族的其他成员如ErbB2/Her2等形成异源二聚体，发生自磷酸化，使其位于胞内的激酶结构域中的磷酸化位点（包括Y992，Y1045，Y1068，Y1148和Y1173等）发生激活，并进一步激活胞内包括Ras/MAPK、PI3K/Akt、JAK/STATs等下游信号通路，参与调控细胞存活、增殖等多个过程。

*EGFR*的突变或过表达与肿瘤的发生密切相关。临床研究发现在肺腺癌细胞中，EGFR经常出现外显子19中的缺失、L858R突变、基因拷贝数增加或者蛋白过表达等现象，造成信号通路的异常激活，进一步引起肿瘤细胞的存活、增殖、侵袭和转移。肺腺癌细胞的存活和增殖依赖EGFR异常活性这一现象的发现（即一种“癌基因依赖”现象），推动了EGFR靶向抗肿瘤药物的研发。目前已经应用于临床的该类药物包括：①单克隆抗体，如cetuximab、panitumumab等，其可以抑制配体与EGFR胞外区的结合，推动受体的内化并引起抗体和补体介导的细胞毒作用；②EGFR-TKI，包括吉非替尼（gefitinib）、厄洛替尼（erlotinib）、克唑替尼（crizotinib）等，其可以通过竞争性结合ATP来抑制EGFR酪氨酸激酶结构域的活性^[5]^（图 1）。尽管EGFR-TKI类药物在临床上对大多数实体肿瘤的治疗效果有限，但可以有效抑制带有EGFR酪氨酸激酶结构域敏感突变位点的NSCLC。最常见的敏感突变包括外显子19密码子746-750附近的非移码缺失突变或外显子21中的L858R突变。目前，此类药物已被用于带有*EGFR*敏感突变位点，特别是不吸烟的亚裔女性肺腺癌患者的临床治疗。

**1:**
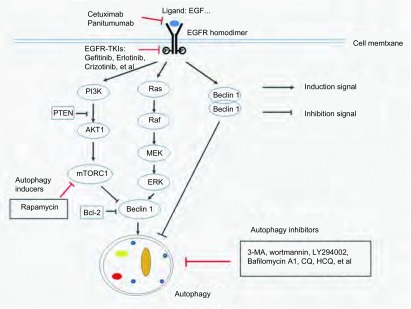
EGFR与自噬的信号通路及相关的药物 Signal pathway between EGFR and autophagy and related drugs. EGFR: epidermal growth factor receptor; CQ: chloroquine; HCQ: hydroxychloroquine

### 1.2 EGFR-TKI类药物耐药机制

在临床研究和应用过程中发现，曾经对EGFR-TKI类药物有很好治疗效果的病例往往在治疗不到一年之后出现普遍的耐药现象，并会在几年之后复发。目前已经确认的造成EGFR-TKI类药物耐药性的机制主要分为四大类^[6]^：①EGFR发生耐药突变。EGFR的T790M突变是最常见的造成EGFR-TKIs耐药的原因^[7]^。该突变会使EGFR上的ATP结合口袋与ATP的结合力增强大约5倍，从而降低了通过竞争结合ATP来发挥作用的EGFR-TKI类药物的效果^[8]^，同时该突变不会影响EGFR的激酶活性^[9]^；其他的此类突变还包括L858R、D761Y、L747S、T854A等^[10-12]^。②“致癌转变”或者旁信号通路的激活。穿膜受体酪氨酸激酶MET由于发生突变、扩增或过表达等原因造成异常活化，以及其配体HGF（hepatocyte growth factor）的过表达，是NSCLC在EGFR-TKI治疗之后发生频率第二高的耐药机制^[13, 14]^；同为HER家族的HER2的过表达或突变也会造成EGFR-TKI的耐药^[15]^；另外，EGFR下游通路活性的异常，主要是*K*-*Ras*发生致癌突变，会表达组成型激活的RAS蛋白，造成gefitinib或erlotinib单药治疗耐药^[16]^。③EGFR-TKI诱导凋亡过程中关键因子的失效。EGFR-TKI类药物在带有敏感突变的肿瘤细胞中诱导凋亡需要促凋亡因子BIM的表达，当BIM由于突变造成其中的BH3结构域（促凋亡BCL2同源结构域3）缺失，便会在慢性粒细胞白血病（chronic myelogenous leukemia, CML）和NSCLC细胞中引起EGFR-TKI的耐药，该耐药可以通过使用BH3模拟药物克服^[17]^。④NSCLC细胞发生上皮-间质转化（epithelial mesenchymal transition, EMT）或转化为SCLC。研究发现EGFR-TKI治疗的病例中会出现间质细胞表型，EMT可能是通过PI3K/AKT通路激活AXL造成的。E-cadherin的下调可以激活EGFR–MEK/ERK/ZEB1/MMP2通路活性，并进一步造成NSCLC的转移侵袭^[18]^，靶向EMT通路中的相关因子可以克服由其造成的耐药^[19]^。另外肿瘤细胞转化成为小细胞癌也是造成耐药的一个原因^[20]^。以上四类机制都已在发生获得性耐药的肺腺癌患者体内检测到。有些机制如T790M突变等，在天然耐药过程中也发挥重要作用^[21]^。近些年，随着对自噬和靶向药物研究的不断深入，发现自噬也可以引起EGFR-TKI的耐药或影响其治疗效果。深入研究造成靶向药物耐药的机制，以及探索有效的抗耐药治疗策略具有重大现实意义。

## 2 细胞自噬及其在肿瘤发生和治疗过程中的作用

### 2.1 自噬及其调控

自噬是细胞的一个受到精确调控的高度保守的过程，其可以在很短时间内实现细胞内容物，包括细胞器和蛋白等的批量降解，使得细胞可以在压力、缺氧和饥饿等情况下存活。自噬在进化上具有高度保守性，广泛存在于从酵母、线虫、果蝇到高等脊椎动物的细胞中。根据细胞内容物进入溶酶体方式的不同，自噬又分为大自噬（macroautophagy）、小自噬（microautophagy）和分子伴侣介导的自噬（chaperone-mediated autophagy）。其由一系列自噬相关基因（autophagy associated gene, ATGs）介导，涉及包括形成自噬体、与溶酶体融合形成自噬溶酶体，以及在水解酶的作用下降解所包裹的细胞器或部分细胞质等一系列动态过程，以实现胞内大分子的循环利用^[22]^。自噬可由饥饿、缺氧、射线照射、生长因子信号抑制剂、传统的化疗和靶向药物等因素诱导^[23]^。研究发现自噬缺陷或异常在神经元病变、微生物感染^[24]^和癌症^[25]^等多种疾病的发生中起到关键作用。

大自噬是肿瘤细胞中出现的主要形式，其典型结构特征是细胞质中出现双层膜空泡结构的自噬体^[26]^。自噬主要包括四个步骤：①吞噬体（phagophore）的形成，这一过程始于一个包含Atg1、Atg13和Atg17的复合物的形成，该复合物会进一步募集膜蛋白Atg9、PI3KⅢ复合物和结合有Beclin1（Atg6）-Vps34的复合物并组装形成吞噬体，对于线粒体自噬（mitophagy），线粒体的降解造成BNIP3[B cell lymphoma 2 (Bcl-2)/adenovirus E1B 19-kDa interacting protein 3]与Bcl-2结合，使得与Bcl-2结合的Beclin1释放，并进一步形成Beclin1-Atg14-PI3K Ⅲ复合物起始自噬；②吞噬体上蛋白的组装，Atg12被Atg7激活，并通过Atg10与Atg5共价结合，Atg12-Atg5的结合推动了吞噬体的延伸和闭合；③自噬体的形成（autophagosome），LC3由Atg4剪切形成LC3-I，并进一步由Atg7和Atg3激活，LC3-I与磷脂酰乙醇胺（phosphatidyl ethanolamine, PE）结合最终形成LC3-Ⅱ-PE复合物，并整合进入吞噬体膜中形成自噬体；④形成自噬溶酶体（autolysosome），自噬体通过与溶酶体融合变成成熟的自噬溶酶体，包入的细胞之中的蛋白或细胞器被溶酶体中的水解酶所降解^[27, 28]^，完成整个自噬过程。

mTOR（mammalian target of rapamycin）和Beclin1-Vps34是自噬调节的中心，许多致癌或肿瘤抑制作用都会影响它们。mTOR是关键的自噬负调控因子，它是复合物mTORC1和mTORC2的组成部分^[29]^。mTORC1对雷帕霉素（rapamycin）敏感，其激活后会进一步激活pS6K1（ribosomal protein S6 kinase 1）和抑制4EBP1（eukaryotic initiation factor 4E-binding protein 1）促进蛋白合成和细胞生长^[30, 31]^。mTORC1与ULK-Atg13-FIP200的结合可以介导自噬的抑制^[32]^。Beclin1-Vps34复合物是另一个重要的自噬调控子，位于mTORC1的下游^[33]^。Beclin1与Vps34结合并激活，对于自噬体的形成非常重要。Beclin1-Vps34由不同的与Beclin1集合的调节蛋白所组成，通过形成不同的复合物在自噬中发挥不同的作用^[34]^。研究发现许多原癌基因和抑癌基因通过直接或间接的调节mTORC1和Beclin1-Vps34对自噬产生不同影响。

### 2.2 自噬和癌症

研究发现自噬在肿瘤发生和发展过程中发挥“双刃剑”的作用，许多研究^[35]^表明自噬有抑制肿瘤的作用，其可以通过清除受损的细胞器、维持细胞的动态平衡、保护正常细胞的生长等作用抑制肿瘤；另外自噬还可以通过引起自噬性细胞死亡抑制肿瘤发生，这一过程不依赖含半胱氨酸的天冬氨酸蛋白水解酶（caspase），在某些凋亡缺陷的情况下是引起肿瘤细胞死亡的重要机制^[36, 37]^。另一方面，自噬是肿瘤细胞在面对各种生长压力时存活的重要机制，当肿瘤细胞遇到营养缺乏等压力时，可以通过快速提高自噬水平，降低生长速度，降解多余或无用的蛋白和细胞器，实现代谢的动态平衡，保证其可以适应微环境的变化并最终存活下来^[38]^。与在肿瘤发生发展中的作用相似，自噬在肿瘤的治疗过程中也发挥着双重作用，造成治疗效果的增强或减弱^[39]^，自噬会在某些肿瘤中介导耐药，这种情况下抑制自噬可以有效的克服抗肿瘤治疗的耐药性^[40]^；然而还有研究^[41]^证明，自噬是治疗过程中介导细胞死亡的重要机制，这种情况下诱导自噬则可以有效提高治疗的效果。自噬在肿瘤发生和治疗过程中的双重作用，使得如何通过系统调节自噬以提高治疗效果变得十分必要却又充满困难，越来越多的研究将自噬作为一个治疗肿瘤的靶点。特异性的使用自噬药物进行调控是一个很有希望的新型治疗方案，可以有效的补充目前使用的肿瘤治疗方案^[42-45]^。

## 3 细胞自噬在EGFR-TKI类药物的治疗和耐药中作用

### 3.1 EGFR和自噬

EGFR与生长因子结合后会造成下游PI3K/AKT、MAPK、Jak/Stat等多条信号通路的激活，进一步通过多种机制参与对自噬的调控。PI3K-I是一个脂类激酶，可以被EGFR磷酸化^[46]^。活化的PI3K-I可以激活Akt1，进一步通过TSC2（tuberous sclerosis protein 2）依赖或非依赖的通路激活mTORC1，同时还可以磷酸化Beclin1并进一步磷酸化Vps34，造成Vps34活性的下降和自噬的抑制^[47]^。另外，Akt介导的Beclin1磷酸化还可以通过形成Beclin 1/14-3-3/vimentin中间丝复合物抑制自噬^[48]^。活化的PI3K-I也可以磷酸化Akt2，其通过AKT2-mTOR-p70S6K通路和抑制线粒体自噬在细胞的增殖和存活中起关键作用^[49]^。另外，EGFR-TKI可以直接磷酸化Beclin1，造成Beclin1发生同源二聚化而抑制Vsp34的活性，进而抑制自噬^[50]^。有研究报道Ras-MEK-ERK（extracellular signal-related kinase）通路在多种肿瘤中都发生失调，其在自噬的调节中发挥双重作用。Ras可以通过激活mTORC1抑制自噬，处于营养缺乏条件下的NIH-3T3细胞中可致癌的HRAS-V12通过激活PI3K-I，进一步通过Akt和mTORC1抑制自噬^[51]^；ERK在自噬中发挥多重作用，一方面，其在体外可以通过促进Beclin1表达和诱导TSC2表达造成mTORC1的失稳诱导自噬^[52]^；另一方面，ERK还可以通过抑制TSC2而激活mTORC1来抑制自噬^[47, 53]^。EGFR还是葡萄糖转运蛋白1（sodium/glucose cotransporter 1, SGLT1）的稳定器，可以增强肿瘤细胞获得能量物质葡萄糖的能力，从而使细胞可以忽略胞外葡萄糖的浓度而存活。EGFR的这一功能可以使胞内保持一定水平的ATP，有效抑制细胞发生凋亡、坏死和自噬性细胞死亡^[54]^。另外，EGFR还可以在恶劣的生长环境中诱导自噬，进一步造成线粒体转位，使肿瘤细胞可以在EGFR-TKI治疗中存活并产生耐药性^[55, 56]^（图 1）。

### 3.2 自噬在EGFR-TKIs治疗和耐药中的作用

天然和获得性耐药是EGFR-TKI对NSCLC治疗失败的主要原因。除了已发现的四大类耐药机制，近些年许多研究^[40]^证实肿瘤细胞还可以通过保持高水平自噬使其在EGFR-TKIs治疗中存活下来。一些研究证明了EGFR-TKIs和自噬抑制剂在耐药细胞中的协同作用。Han等^[23]^发现吉非替尼和厄洛替尼可以在表达野生型EGFR的耐药NSCLC中通过抑制PI3K/AKT/mTOR信号通路诱导高水平自噬，而在EGFR-TKI敏感的细胞中则不会诱导明显的自噬，耐药细胞中药物诱导的细胞毒性作用在抑制自噬之后得到明显增强。Zou等^[57]^发现厄洛替尼可以在表达野生型EGFR的耐药NSCLC中诱导自噬，当厄洛替尼和自噬抑制剂氯喹（chloroquine, CQ）或羟氯喹（hydroxychloroquine, HCQ）共同使用时肿瘤生长的抑制效果会得到明显加强，进一步分析发现肿瘤细胞中凋亡水平明显增强，但是细胞周期以及EGFR下游信号通路活性则没有明显变化。Sakuma等^[58]^发现EGFR突变的肺腺癌吉非替尼耐药细胞中，包括发生EMT的细胞亚群，细胞的存活不依赖于EGFR的活性，而是可以通过诱导持续的自噬在缺氧环境中存活下来，用siRNA敲除Atg5或使用CQ抑制自噬可以明显降低该细胞在缺氧条件下存活的能力。Nihira等^[59]^报道LC3A介导的自噬激活在肺腺癌细胞的厄洛替尼耐药性中发挥重要作用，抑制LC3A或自噬可以恢复耐药株对药物的敏感性，另外还发现肺腺癌患者肿瘤中LC3A的表达水平与EGFR-TKI治疗后的存活率呈负相关。Lee等^[60]^也发现厄洛替尼耐药的NSCLC比过表达EGFR的敏感细胞的自噬水平更高，自噬抑制剂3-甲基腺嘌呤（3-methyladenine, 3-MA）可以使耐药株恢复对药物的敏感性，而自噬诱导剂rapamycin则可以进一步增强细胞的耐药性。Li等^[61]^的结果显示厄洛替尼可以在敏感的NSCLC中诱导自噬，这一过程与p53的核定位、AMPK的激活和mTOR的抑制有关，用siRNA敲除Atg5和Beclin1或使用CQ抑制自噬可进一步增强厄洛替尼对敏感细胞的生长抑制作用；但是，厄洛替尼却不能在耐药的NSCLC细胞中诱导自噬。Bokobza等^[62]^的研究证明同时使用Akt抑制剂CQ和EGFR-TKI可以增强对EGFR-TKI敏感的NSCLC细胞的生长抑制作用。Wang等^[63]^报道厄洛替尼可以在敏感和耐药细胞中诱导自噬，抑制自噬可以增强对NSCLC的杀伤作用，这一过程是通过内质网应激（endoplasmic reticulum stress, ER stress）的CHOP蛋白介导的。另外，在其他携带EGFR过表达突变的肿瘤，如乳腺癌^[64]^，质母细胞瘤^[65]^，头颈鳞状细胞癌^[66]^等中也有相似的报道。这些发现都支持自噬是一个细胞存活机制，造成NSCLC对EGFR-TKI的耐药，抑制自噬可以增强肿瘤细胞对药物敏感性。

与此同时，Fung等^[67]^的研究表明自噬的缺失可能是一个造成EGFR-TKI耐药的机制，这种情况下，联合使用EGFR-TKI和自噬诱导剂可以提高治疗效果。尽管在多种肿瘤细胞中自噬水平与EGFR-TKI存在剂量依赖关系，高度耐药细胞中自噬并没有明显的激活，雷帕霉素能够增加细胞对药物的敏感性，并且敲除关键的自噬基因Atg7会进一步抑制药物的敏感性。Gorzalczany等^[68]^发现在表达野生型EGFR的耐药NSCLC中，雷帕霉素可以增加细胞对药物的敏感性，这一作用与自噬水平的增加和线粒体膜电位的超极化有关。Xu等^[69]^发现依维莫司（RAD001，mTOR抑制剂）可以通过增强吉非替尼诱导的自噬来增加后者对肺癌H460的生长抑制作用，这一过程与AMPK的激活有关。La Monica等^[70]^的研究也有相似的结果，依维莫司可以增加耐药NSCLC对EGFR-TKI的敏感性，同时使用依维莫司和吉非替尼会引起MAPK和mTOR以及EGFR信号活性的明显下降，进一步抑制细胞的生长。另外，Schmid等^[71]^发现同时使用厄洛替尼和依维莫司可以抑制EGFR和mTOR的协同效果，有效抑制SCLC，RAD001通过抑制mTOR诱导自噬，该抑制效果与DNA合成的降低、细胞周期的G0/G1捕获和诱导自噬有关。Wei等^[50]^证明活化的EGFR可以直接磷酸化Beclin1，并进一步通过造成Beclin1与抑制子结合最终抑制自噬，这一过程与NSCLC的进展和耐药有关，EGFR-TKI可以通过抑制这一过程使细胞发生自噬来发挥作用，这一结果证明在接受该类靶向药物治疗的患者中使用自噬抑制剂可能会对临床效果产生不利的影响。

目前已经开展了几项研究CQ和HCQ与EGFR-TKI联合治疗效果的临床实验。一项I期临床实验研究在晚期NSCLC病例中联合使用HCQ与厄洛替尼的安全性、最高剂量、临床反应和药物动力学^[72]^。另外还有评价NSCLC患者中HCQ联合吉非替尼以及其他化疗药物治疗效果的临床实验^[73]^。研究诱导自噬在肿瘤靶向治疗过程中效果的临床实验目前还没有报道。对于评价自噬调节药物和EGFR-TKI联合使用的效果，还需要进一步展开更多系统性的临床实验。

## 4 总结和展望

自噬在肿瘤的发生发展和治疗过程中的作用无疑是复杂的，发挥着“双刃剑”的作用：既可能是抑制肿瘤的机制，又可能是肿瘤细胞存活的机制，以相反的方式影响抗肿瘤治疗的效果。研究自噬在肿瘤的发生发展和治疗过程中的功能，以及各类自噬调控药物的功能和应用，为实现癌症的治疗提供了新的机会。

同时，EGFR-TKIs作为研究的最透彻、最为成熟的肿瘤靶向药物也被寄予厚望。EGFR-TKI和西妥昔单抗等可以诱导肿瘤细胞发生自噬，但是自噬的作用同样是两方面：一些研究证明EGFR-TKI诱导的自噬是肿瘤细胞的一个保护机制，自噬抑制剂可以增强药物的细胞毒性效果；而还有研究证明EGFR-TKI诱导的高水平自噬可以在凋亡缺陷的肿瘤细胞中造成自噬性死亡，这种情况下联合使用自噬诱导剂则可能产生更好的效果。

未来的研究应该集中在以下方面：①开发新的研究自噬的模型，在不同的肿瘤和治疗阶段中研究联合使用EGFR-TKI和自噬调节药物的使用策略；②研究自噬在EGFR抑制作用中作为细胞死亡或存活作用的具体机制，以及EGFR在自噬调节过程中的作用；③随着新的自噬调节药物不断涌现，需要系统的开展更多的EGFR-TKI联合自噬调节剂治疗方案的临床实验；④EGFR对自噬的调控在推动肿瘤进展和耐药中的作用也需要进一步的探索。
